# Metabolic Enzyme MeHNL11 Regulates *MeCAS1b* Transcription for Cyanide Reutilization in Response to Nitrate Deficiency in Cassava

**DOI:** 10.1111/pbi.70633

**Published:** 2026-03-10

**Authors:** Weitao Mai, Ruxue Bao, Xiaocheng Liu, Mengtao Li, Jinling Zhao, Huaifang Zhang, Yuan Yao, Haiyan Wang, Wenquan Wang, Changying Zeng, Xin Chen

**Affiliations:** ^1^ Sanya Institute of Breeding and Multiplication/School of Tropical Agriculture and Forestry State Key Laboratory of Tropical Crop Breeding, Hainan University Sanya Hainan China; ^2^ Institute of Tropical Bioscience and Biotechnology Chinese Academy of Tropical Agricultural Sciences Haikou Hainan China; ^3^ Sanya Research Institute Chinese Academy of Tropical Agricultural Sciences Sanya Hainan China

**Keywords:** bifunctional protein, cyanogenic glucosides, detoxification of cyanide, low nitrate stress, nitrogen reuse, nucleus translocation, primary nitrogen metabolits, transcriptional regulation

## Abstract

Cassava ( *Manihot esculenta* Crantz) exhibits exceptional tolerance to infertile soils and contains abundant cyanogenic glucosides (CGs). Previous research has indicated that CGs can serve as a significant reservoir of organic nitrogen in plants. However, the extent to which its high‐CG content contributes to efficient nitrogen utilisation and adaptation to low nitrogen (N) in cassava remains to be further elucidated. This study represents the first identification of MeHNL11 as a bifunctional protein. In response to N deficiency, the hydroxynitrile lyase activity of MeHNL11 promotes the generation and accumulation of cyanide and the Cys245 residue of MeHNL11 is critical for its nuclear oligomerization, in which the protein functions as a transcription factor. Following the cyanide transmission into the nucleus, the oligomeric form of MeHNL11 dissociates into monomers, leading to a dramatic upregulation of *MeCAS1b* transcription. This regulatory mechanism helps sustain intracellular cyanide homeostasis within cassava and facilitates the synthesis of primary N metabolites, thereby alleviating N deficiency. The exogenous application of the cyanide antidote hydroxocobalamin (COB) inhibited cyanide assimilation by MeCAS1b, leading to exacerbated N deficiency symptoms, such as leaf yellowing and a significant reduction in the contents of NH_4_^+^ and free amino acids (AA) in cassava seedlings under low‐N conditions (LN). Our research demonstrates that the MeHNL11‐*MeCAS1b* module plays a pivotal role in CG recycling, offering new insights into the underlying mechanisms governing cassava's exceptional tolerance to low N stress.

## 1 Introduction

Cassava ( *Manihot esculenta* Crantz) is a vital staple crop for approximately 800 million people and ranks as the sixth largest food crop globally. It exhibits remarkable adaptability to low soil fertility. The apparent nitrogen (N) utilisation rate of cassava can reach 50%–70% (Adiele et al. 2020), which is considerably higher than that of conventional cereal crops. However, the molecular mechanisms underlying its tolerance to low N conditions are still largely unexplored.

N use efficiency (NUE) is a complex agronomic trait influenced by three critical internal processes: N absorption, assimilation, and remobilization (Hu et al. 2023). Plants primarily acquire inorganic N from the environment and convert it into organic forms to support their growth. Upon N deficiency, plants can fulfil their growth requirements by promoting N uptake through their roots or reusing N reserves stored within their tissues (Wang et al. 2024). Plant secondary metabolites have been identified to play roles in nutrient recycling, such as glucosinolates, which are catalysed and reintegrated into primary sulfur metabolism in *Arabidopsis* (Sugiyama et al. 2021). However, whether N‐containing secondary metabolites can contribute to N reuse under N‐limited conditions remains unclear.

Cyanogenic glycosides (CGs) are natural plant defence compounds synthesised by the condensation of the hydroxyl base of cyanohydrin derivatives with D‐glucose. They are distributed across various cyanogenic plant families, such as Leguminosae, Rosaceae and Asteraceae (Sánchez‐Pérez and Neilson 2024). Cassava, renowned for its high content of CGs, primarily contains two aliphatic monoglycosides: linamarin (95%) and lotaustralin (5%) (Siritunga and Sayre 2003). The biosynthetic pathway for CGs in cassava has been well characterised, with the rate‐limiting step involving the conversion of valine and isoleucine to acetaldoxime—a reaction catalysed by cytochrome P450 monooxygenases CYP79D1/D2 (Andersen et al. 2000). Typically, CGs are sequestered in vacuoles and are physically separated from their catabolic enzymes. However, when herbivores ingest cassava, CGs are decomposed into acetone and the highly toxic substance hydrocyanic acid through the sequential action of cell wall β‐glucosidase (BGLUs) and hydroxynitrile lyases (HNLs) (White et al. 1998). Plants have evolved a mechanism to assimilate HCN through the cyanoalanine pathway, where HCN and cysteine are converted into hydrogen sulfide (H_2_S) and cyanoalanine by the action of cyanoalanine synthase (CAS). Cyanoalanine is subsequently metabolised by nitrilase (NIT4) and converted into asparagine/aspartic acid and ammonium (Siegień and Bogatek 2006). This metabolic process alleviates the toxicity of hydrocyanic acid and incorporates it into nitrogenous primary metabolites. Hydrogen cyanide is a weak acid with a pKa of 9.2. At physiological pH, it exists predominantly in the undissociated form (HCN, ~95%), with the remainder (~5%) as the dissociated cyanide anion (CN^−^) (Zuhra et al. 2025). Throughout this paper, both forms are collectively referred to as ‘cyanide’.

Beyond its crucial role in HCN detoxification, CAS significantly regulates plant growth, development and stress adaptation by maintaining the balance between HCN and H_2_S within plants. The *atcas‐c1* mutant exhibited an accumulation of cyanide and a distinctive shortening of root hair, which could be restored either by complementation with the wild‐type *AtCAS‐C1* gene or by treatment with hydroxocobalamin (COB), a known cyanide antidote (García et al. 2010; Arenas‐Alfonseca et al. 2018). Moreover, the overexpression of *CAS* was shown to alleviate the excessive accumulation of cyanide and reactive oxygen species (ROS), thereby enhancing plants' salt tolerance (Yu et al. 2021; Xu et al. 2023). Moreover, AtCAS‐C1 has been identified as a mitochondrial H_2_S donor and is involved in the stomatal closure induced by the bacterial flagellin peptide flg22 (Zhou and Xie 2024). These findings highlight the multifaceted roles of CAS in plant physiology.

Hydroxynitrile lyase (HNL) has been identified across a variety of plant species, including *Arabidopsis*, cassava, almond, flax and rubber tree. As a biocatalyst, HNL can reversibly catalyse the formation of optically active cyanohydrins from HCN and aldehydes or ketones (Chueskul and Chulavatnatol 1996). Overexpression of *AtHNL* has been shown to increase cyanide content and enhance resistance to mite infestations (Arnaiz et al. 2022). In cassava, *MeHNL* overexpression led to an 80% reduction in CG content, a 22% increase in free amino acid content and a threefold elevation in protein content in roots (Narayanan et al. 2011). These findings suggest that manipulating *HNL* expression could be a promising strategy for improving cassava's nutritional quality and safety.

Under N deficient conditions, cyanide can serve as an alternative N source for plants, highlighting a fascinating aspect of plant nutrient acquisition. When ammonium (NH_4_^+^) and cyanide are applied simultaneously to wheat and sorghum, a decrease in NH_4_^+^ supply notably enhances the transport and assimilation of cyanide, supporting normal plant growth (Ebbs et al. 2010). Furthermore, in N‐deprived environments, exogenous application of cyanide stimulates the activities of key enzymes such as cysteine synthase (CAS) and asparaginase, leading to improved plant growth compared to untreated plants (Li et al. 2020; Liu et al. 2020). These evidences indicate that plants can absorb exogenous cyanide during periods of N deficiency.

In this study, we identified that *MeCAS1b* was significantly upregulated in response to low N stress. Notably, treatment with a cyanide antidote resulted in a significant decrease in NH_4_^+^ and AA contents, leading to the manifestation of more severe N deficiency symptoms under N starvation conditions. Interestingly, we discovered that MeHNL11, an upstream metabolic enzyme of MeCAS1b, could bind to the promoter of *MeCAS1b* both in vivo and in vitro. MeHNL11 catalyses the production of HCN, which then induces MeHNL11 to act as a transcription factor, directly upregulating *MeCAS1b* expression to enhance cyanide assimilation. These results indicate that MeHNL11 has the potential to act as a bifunctional gene. Its enzyme activity accelerates the decomposition of CGs while its transcription factor activity promotes the reutilization of cyanide, thereby enhancing N use efficiency in cassava under LN.

## 2 Results

### 2.1 *MeCAS1b* Was Significantly Upregulated in Fibrous Roots Under Low N Conditions

**FIGURE 1:**
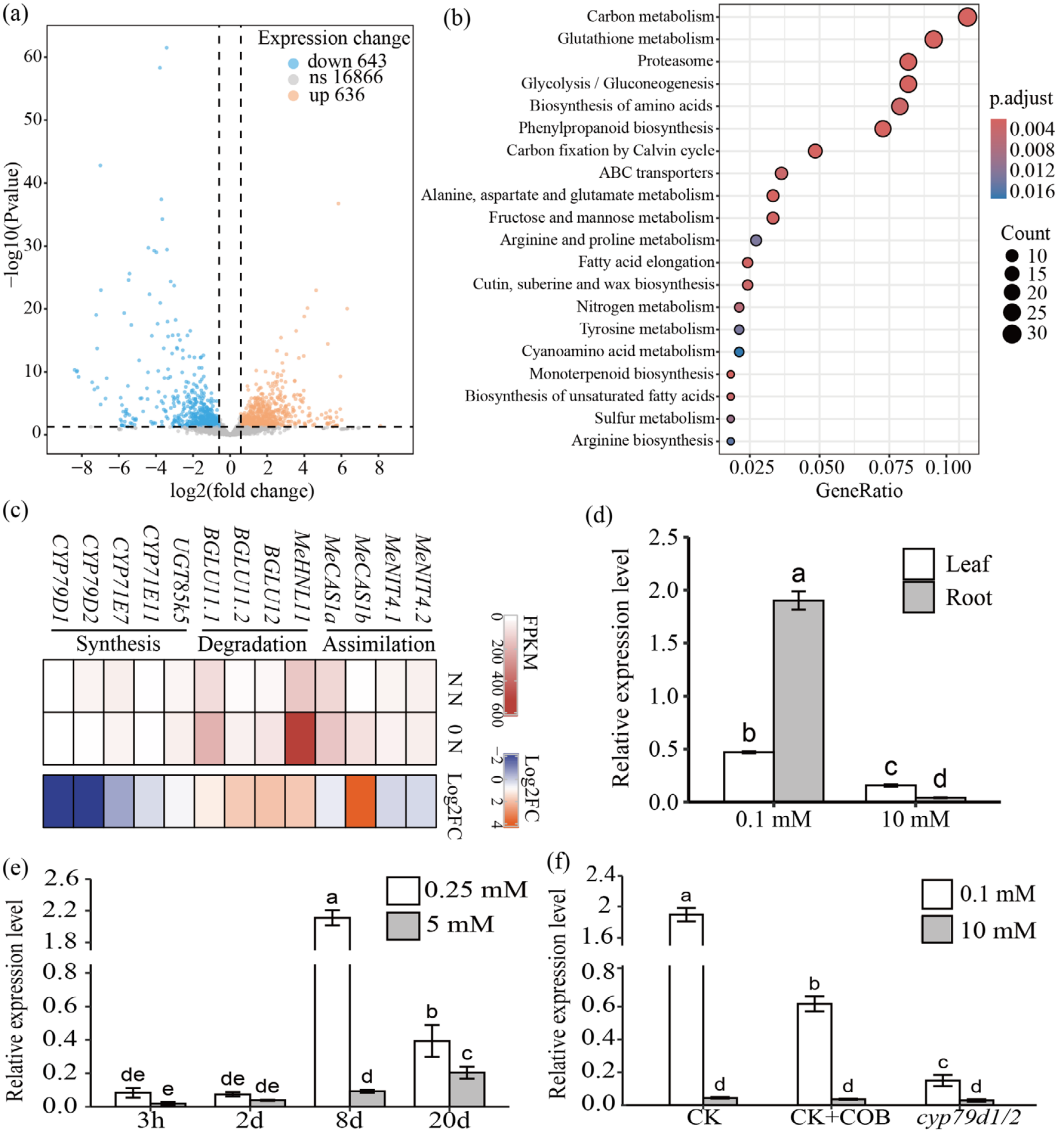
RNA‐seq and transcriptional activity analysis of *MeCAS1b* under different NO_3_^−^ treatments. (a) Volcano plot displayed the fold change versus the *q* value for inter‐group difference analysis. Genes with significant changes were marked with blue and orange dots (*q* < 0.05, FC > 1.5). (b) Functional annotation of differentially expressed genes by KEGG enrichment analysis. (c) Heatmaps illustrated the FPKM values of genes involved in the Synthesis, Degradation and Assimilation of cyanogenic amino acid metabolism pathway. NN: Normal Nitrogen (5 mM NO_3_^−^). 0 N: Nitrogen‐free (0 mM NO_3_^−^). Log2 FC (fold change), the Log2‐transformed value of the ratio of FPKM values under 0 N to those under NN (0 N FPKM)/(NN FPKM). (d) The expression levels of *MeCAS1b* in leaves and roots after 7 days of treatment with 0.1 mM NO_3_^−^ and 10 mM NO_3_^−^. (e) The expression levels of *MeCAS1b* in leaves after 3 h, 2 days, 8 days and 20 days treatments with 0.25 mM NO_3_^−^ and 5 mM NO_3_^−^. (f) The expression levels of *MeCAS1b* in CK (SC8), CK + COB (SC8 + 5 mM COB) and *cyp79d1/d2* under treatments with 0.1 mM NO_3_^−^ and 10 mM NO_3_^−^. The data are presented as the mean ± SE (*n* ≥ 3). a/b/c/d indicates statistically significant differences at *p* < 0.05.

RNA‐seq analysis was conducted on the roots of cassava SC8 (South China 8) plantlets subjected to either N deficiency (0 mM) or normal N levels (5 mM NO_3_^−^ or 5 mM NH_4_^+^) for 2 h. After filtering low‐quality data and clean reads alignment analysis, 1279 differentially expressed genes were identified, including 636 genes upregulated and 643 genes downregulated (Figure 1a). The KEGG enrichment analysis revealed that these differentially expressed genes were primarily involved in carbon metabolism, N metabolism and glutamine metabolism (Figure 1b). Notably, the cyanogenic amino acid metabolism pathway, which encompasses both the synthesis and degradation of cyanogenic glycosides as well as the assimilation of cyanide, was highly enriched. Specifically, the key gene *MeCYP79D2*, which plays a crucial role in the synthesis of cyanogenic glycosides, was significantly downregulated (Figure 1c). In contrast, *MeHNL11*, which is responsible for the decomposition of cyanogenic glycosides, along with *MeCAS1b*, its enzyme involved in cyanide base assimilation, were both significantly upregulated under low N conditions (LN) compared to normal N conditions (NN) (Figure 1c). RT‐qPCR analysis verified that *MeCAS1b* was significantly upregulated in both leaf and root under LN conditions compared to NN. Interestingly, this gene exhibited the highest transcriptional activity in roots under LN, while predominantly expressed in leaves under NN (Figure 1d). Following an N‐deficient treatment of 0.1 mM NO_3_^−^ for 8 days, there was a most pronounced increase in the expression level of *MeCAS1b* in the roots (Figure 1e). The *cyp79d1/d2* mutants and exogenous COB application, along with CK were further subjected to LN (0.1 mM) and NN (10 mM) treatment for 7 days. The results reveal that the transcription level of *MeCAS1b* increased by 47.5 fold under N‐deficient treatment, while it only increased by 22.6 fold following additional treatment with cyanide antidote COB. It was observed that the expression level of *MeCAS1b* in the CG‐deficient mutant *cyp79d1/d2* did not show significant changes, with only a 3.4 fold increase (Figure 1f). These results further support that low N induces the up‐regulation of *MeCAS1b* and that up‐regulation positively correlated with the HCN concentration in vivo.

### 2.2 The Block of Cyanide From the Degradation of CG Can Further Exacerbate N Deficiency Symptoms in Cassava

**FIGURE 2:**
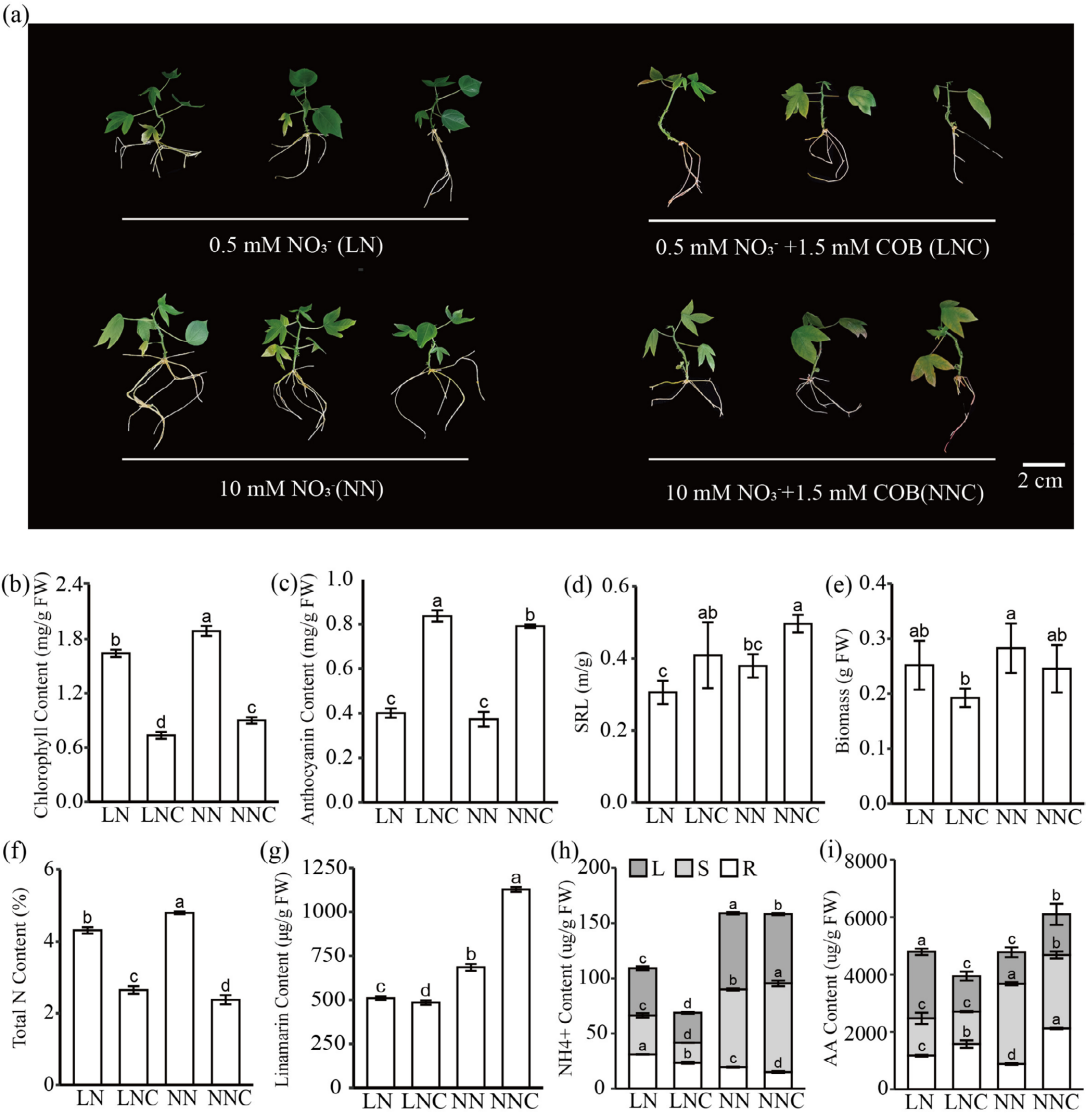
The phenotypic comparison after SC8 treated with COB under low and normal nitrate conditions. (a) The phenotypes of SC8 after 14 days of treatment with 0.5 mM NO_3_^−^ (LN), 0.5 mM NO_3_^−^ + 1.5 mM COB (LNC), 10 mM NO_3_^−^ (NN) and 10 mM NO_3_^−^ + 1.5 mM COB (NNC). Determination of physiological indicators associated nitrogen use efficiency, such as chlorophyll content (b), anthocyanin content (c), specific root length (SRL) = root length/root fresh weight (d), Biomass (e), total nitrogen (f), Linamarin content (g), ammonium nitrogen (NH_4_^+^) (h) and free amino acids (AA) Content (i).

To further investigate the contribution of CGs to cassava's low‐N (LN) adaptation. COB serves as a well‐known antidote for cyanide, was applied under LN and normal‐N (NN) conditions (denoted as LNC and NNC, respectively) to block the assimilation of cyanide by MeCAS1b in cassava plantlets. Under LN, exogenous COB applied plants exhibited more typical N‐deficiency symptoms, including more severe leaf yellowing, thinner and longer roots, lower chlorophyll content, higher accumulation of anthocyanins and lower biomass, which can be anticipated if the treatment time is prolonged (Figure 2a–e). Moreover, the total N content in LNC and NNC was substantially lower than that in LN and NN (Figure 2f), respectively. The CGs content in LNC and LN was significantly lower than that in NNC and NN, respectively (Figure 2g). The NH_4_^+^ and free amino acid (AA) contents in LNC were markedly lower than those in LN, and they were mainly distributed in roots (Figure 2h,i). These findings suggest that a decrease in cyanide concentration reduces the likelihood of cyanide being recycled, leading to more pronounced symptoms of N deficiency in plants. Concurrently, there is a decline in total N content, as well as in the levels of readily mobile N forms such as ammonium ions and free amino acids, along with a reduction in biomass. In summary, cassava facilitates the decomposition of CGs and transforms cyanide into NH_4_^+^ and AA to promote N reuse and enhance adaptation to low N conditions.

### 2.3 Isolation and Bioinformatic Analysis of MeHNL11 in Cassava

We utilised the 860 bp promoter of *MeCAS1b* as bait to screen the cDNA library of cassava roots that had undergone low N treatment. After rigorous double‐checking and PCR identification (Figure S1), 20 candidate interacting proteins were identified by Sanger sequencing and functional annotation (Table S1). Among these candidates, MeHNL11 was remarkable, having the highest screening frequency, appearing 8 times. Hydroxynitrile lyase is responsible for the release of cyanide by decomposing cyanohydrins. Subsequently, its downstream MeCAS1b can assimilate the cyanide into β‐cyanoalanine. This interaction implies a potential regulatory link between MeHNL11 and *MeCAS1b* in the CGs metabolism pathway under N stress conditions.

**FIGURE 3:**
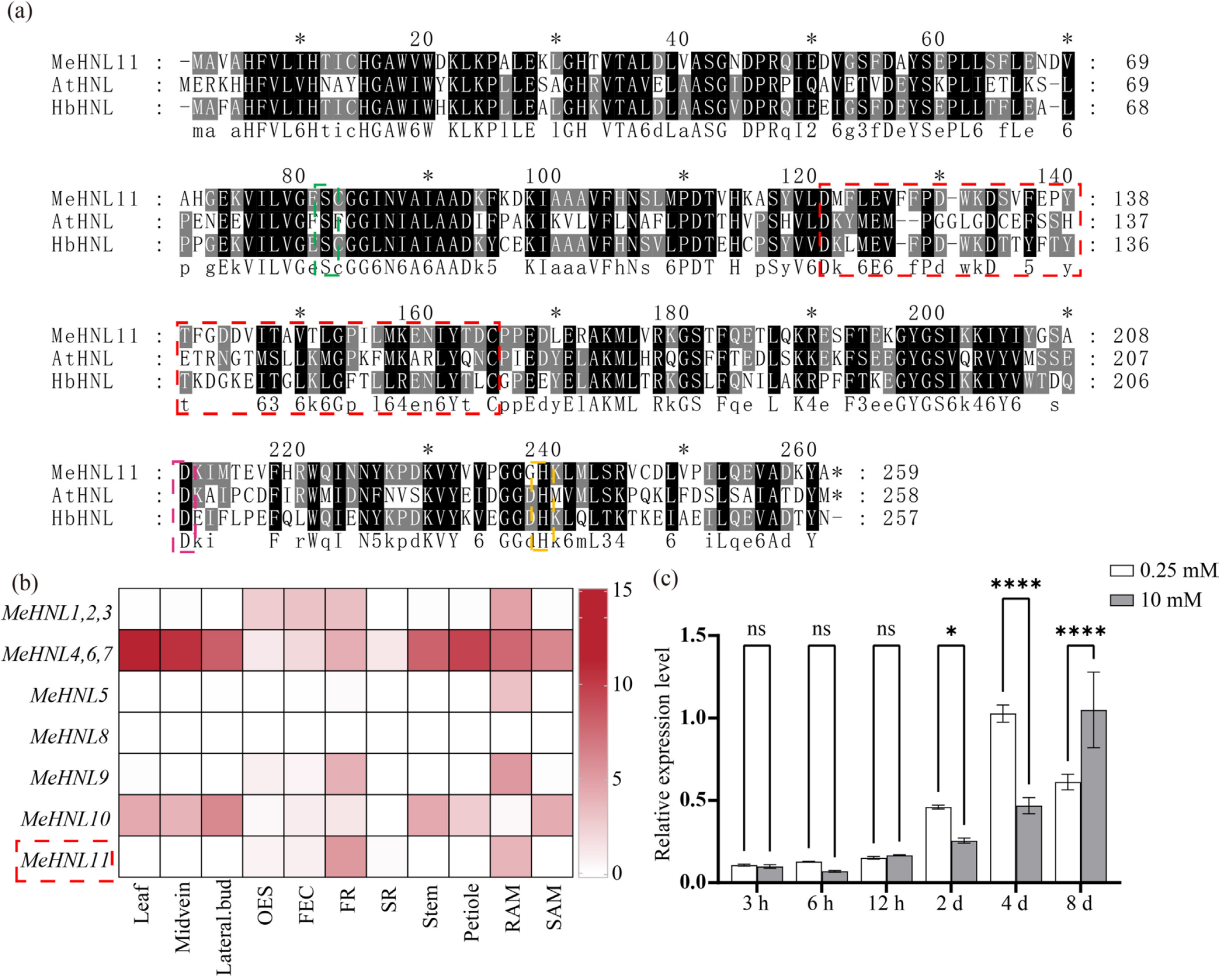
The identification of *MeHNL11* responsive to low nitrate. (a) Comparative homology assessment among *MeHNL11*, *AtHNL*, and *HbHNL*; (b) Analysis of the expression patterns of the MeHNL gene family in 11 cassava tissue which data extracted from Wilson et al. (2017); FEC, Friable callus; FR, Fibrous root; OES, Somatic embryo; RAM, Root apex; SAM, Stem apex; SR, Storage root. (c) The expression levels of *MeCAS1b* in leaves during the periods after 0 h, 3 h, 2 days, and 8 days under treatments with 0.25 mM NO_3_^−^ and 5 mM NO_3_^−^. Significance is denotes as *p* ≥ 0.05 (ns), *p* < 0.05 (*) and *p* < 0.0001 (****).

Among the 11 members of the *HNL* family in cassava, MeHNL11 shows a high level of amino acid sequence homology with its counterparts in *Arabidopsis* and rubber HNL (Figure 3a). It features a conserved peptide substrate recognition and binding site spanning amino acid positions 121–165, along with three catalytic active sites defined by a serine at position 82, an aspartic acid at position 208 and histidine at position 236 (Figure 2a). In other words, the structural domains of HNL imply that MeHNL11 is a classic hydroxynitrile lyase with catalytic activity. According to the 11 tissue‐specific transcriptome analysis of cassava (Wilson et al. 2017), *MeHNL11* (Manes.13G092900) is specifically expressed in roots (Figure 3b). Meanwhile, our RT‐qPCR analysis showed that this gene was significantly upregulated under LN conditions (Figure 3c).

### 2.4 MeHNL11 Shuttled Into the Nucleus From Cytoplasm Under Low N Treatment

**FIGURE 4:**
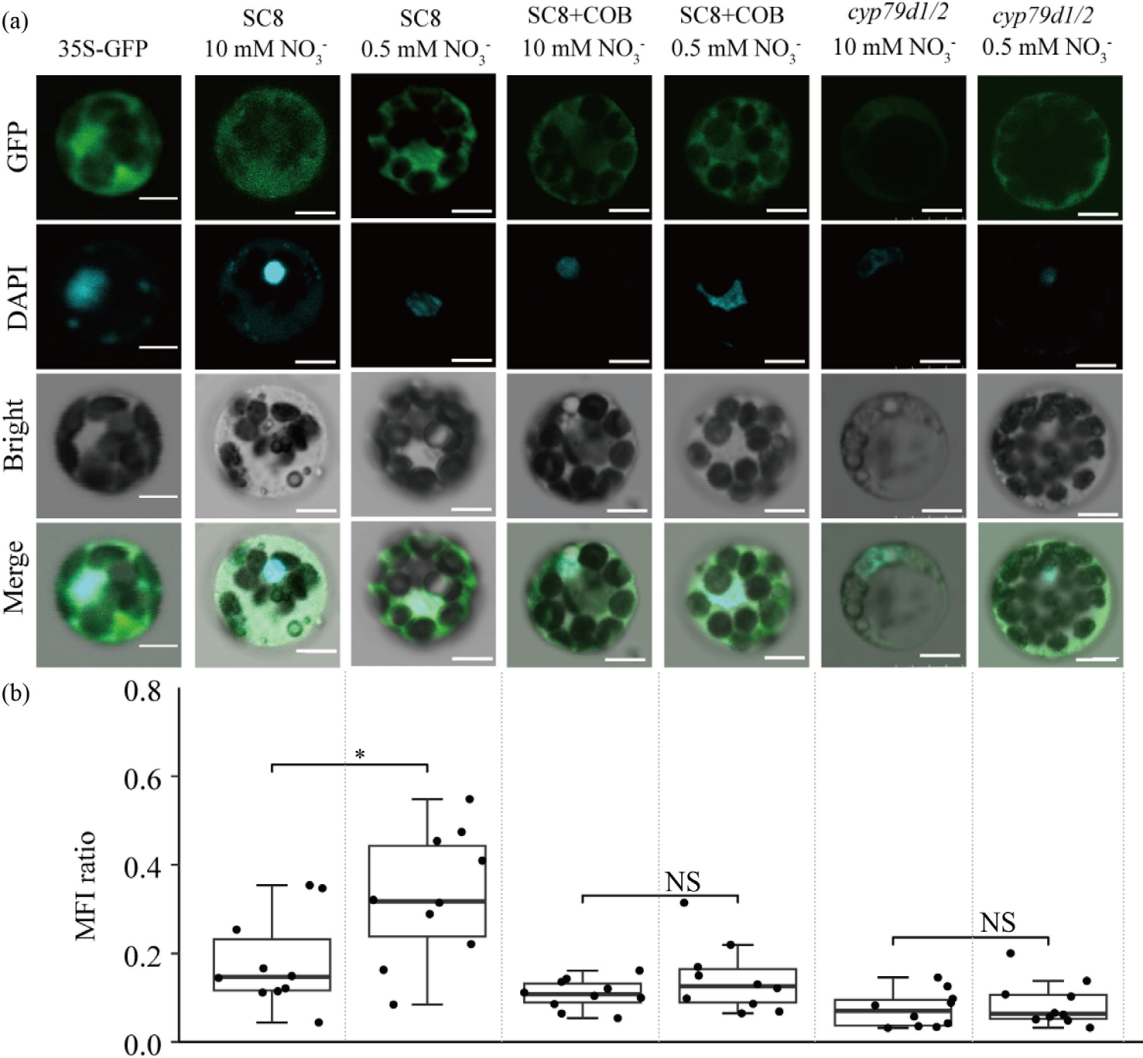
Subcellular localisation of MeHNL11 under low and normal nitrate conditions. (a) Subcellular localisation of MeHNL11 in SC8, SC8 with COB treatment and *cyp79d1/2* protoplasts under NO_3_^−^ treatment. COB concentration: 5 mM, Scale bar: 5 μm. (b) Mean Fluorescence Intensity (MFI) ratio of MeHNL11 in protoplasts. The MFI ratio was calculated as the formula: MFI ratio = the fluorescence intensity in nuclear to that in cytoplasmic area per cell. Significance is denotes as *p* ≥ 0.05 (NS) and *p* < 0.05 (*).

The MeHNL11‐GFP fusion protein, driven by the 35S promoter, was transiently expressed in cassava leaf protoplasts. To quantify the distribution of the protein between the cytoplasm and the nucleus, the mean fluorescence intensity (MFI) ratio of fluorescence was calculated using ImageJ software, with DAPI as a reference. In the protoplasts, the green fluorescence intensity and MFI ratio exhibited a substantial increase when treated with 0.5 mM NO_3_^−^ compared to those treated with 10 mM NO_3_^−^ (Figure 4). Nevertheless, when COB was additionally applied, there was no significant difference in the green fluorescence intensity and MFI ratio between 0.5 mM NO_3_^−^ and 10 mM NO_3_^−^ treatment. Similarly, in the *cyp79d1/d2* mutants, no significant changes were observed in the green fluorescence intensity and MFI ratio between 0.5 mM NO_3_^−^ and 10 mM NO_3_^−^ (Figure 4).

### 2.5 MeHNL11 Has Transcriptional Activation Ability and Can Form Oligomers in the Nucleus

**FIGURE 5:**
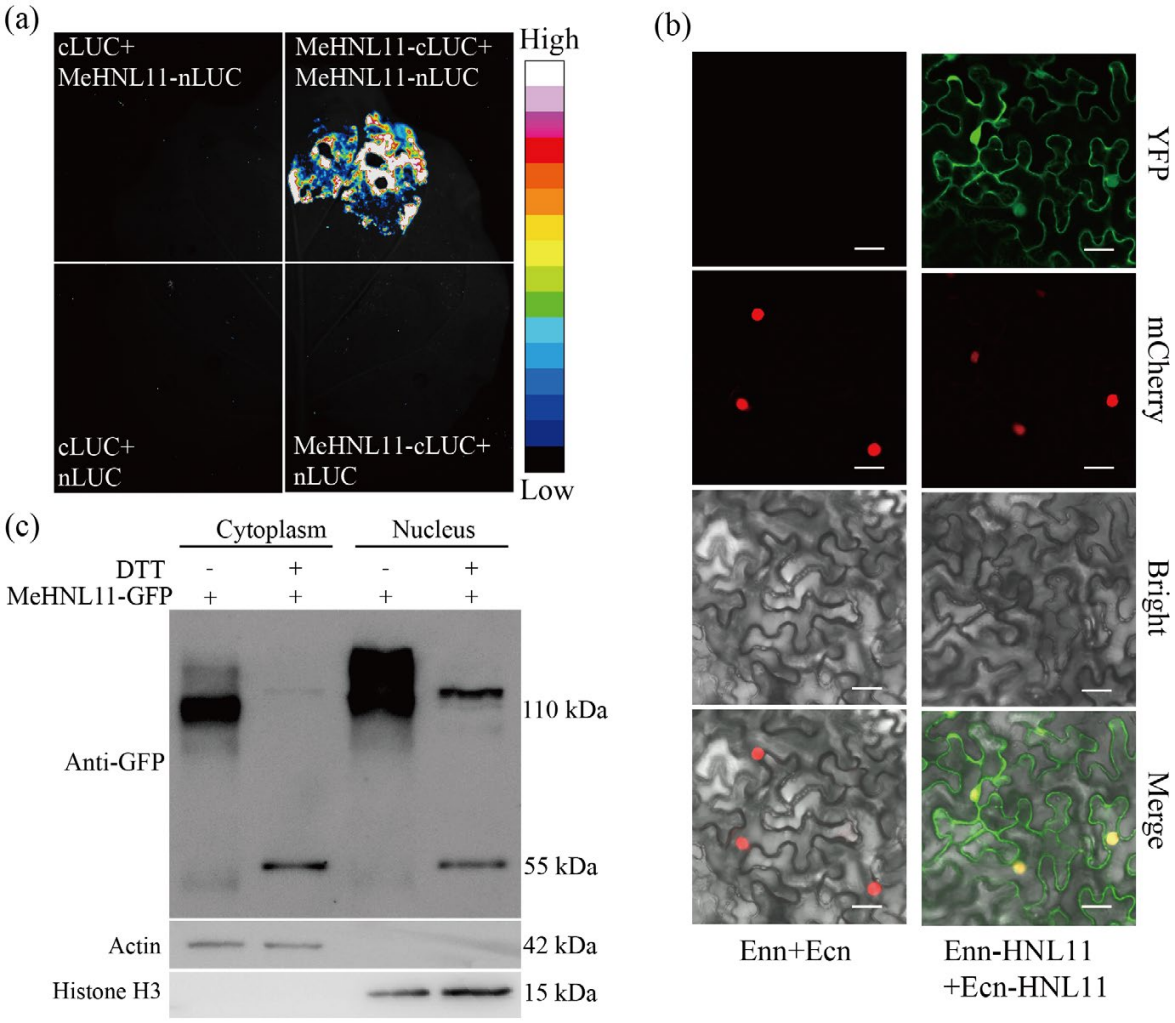
The characteristics of MeHNL11 as a transcription factor. (a) Luciferase complementation imaging (LCI) assays showing the interaction between MeHNL11‐nLUC and cLUC‐MeHNL11 in *Nicotiana benthamiana* leaves. (b) The homodimers forming from Enn‐HNL11 and Ecn‐HNL11 by BiFC assay. Scale bar = 50 μm. (c) Polymerisation forms of MeHNL11‐GFP in cytoplasm and nucleus and the effects of DTT treatment. DTT, Dithiothreitol. Actin and Histone H3 (H3) were used as loading controls.

When MeHNL11 was fused to both the N‐terminal and C‐terminal of LUC, firefly fluorescence was detected in tobacco leaves coinfected with MeHNL11‐cLUC and MeHNL11‐nLUC using the Firefly Luciferase Complementation Imaging assay (LCI) (Figure 5a). Meanwhile, the bimolecular fluorescence complementation assay also demonstrated that MeHNL11 could form homodimers and multimers (collectively designated as oligomers), and these oligomers were distributed in both the nucleus and the cytoplasm (Figure 5b). Furthermore, polyacrylamide gel electrophoresis (PAGE) demonstrated that the MeHNL11‐GFP fusion protein existed as a homodimer (110 kDa) in the cytoplasm. When treatment with strong reductant dithiothreitol (DTT) to disrupt the disulfide bonds, nearly all dimers were dissociated into monomers (55 kDa) (Figure 5c). What's more, within the nucleus, the MeHNL11‐GFP fusion protein was observed in the form of oligomers (≥ 110 kDa). After DTT treatment, only a fraction of the MeHNL11‐GFP proteins were depolymerized into monomers (55 kDa) (Figure 5c). Collectively, these results indicate that MeHNL11 can form oligomers via disulfide bonds.

On one hand, our above results have demonstrated that low N treatment can trigger MeHNL11 to translocate into the nucleus, where it forms oligomers. On the other hand, MeHNL11 has been proven to bind to the *MeCAS1b* promoter through the yeast one‐hybrid (Y1H) assay. Combining these findings, it is reasonable to speculate that MeHNL11 can not only function as a hydroxynitrile lyase but also play a role in regulating the transcription of its downstream genes. Consequently, the transcriptional activation ability of MeHNL11 was investigated using the yeast two‐hybrid (Y2H) assay. The results showed that Y2H strains co‐transfected with PGBKT7‐MeHNL11 and pGADT7 thrived on selective media (SD/‐TA, SD/‐TH) and turned blue on medium containing X‐α‐Gal (Figure S2). This confirmed that MeHNL11 has the capacity to activate transcription.

### 2.6 MeHNL11 Binds to the *MeCAS1b* Promoter and Activates the Transcription Activity of *MeCAS1b*

**FIGURE 6:**
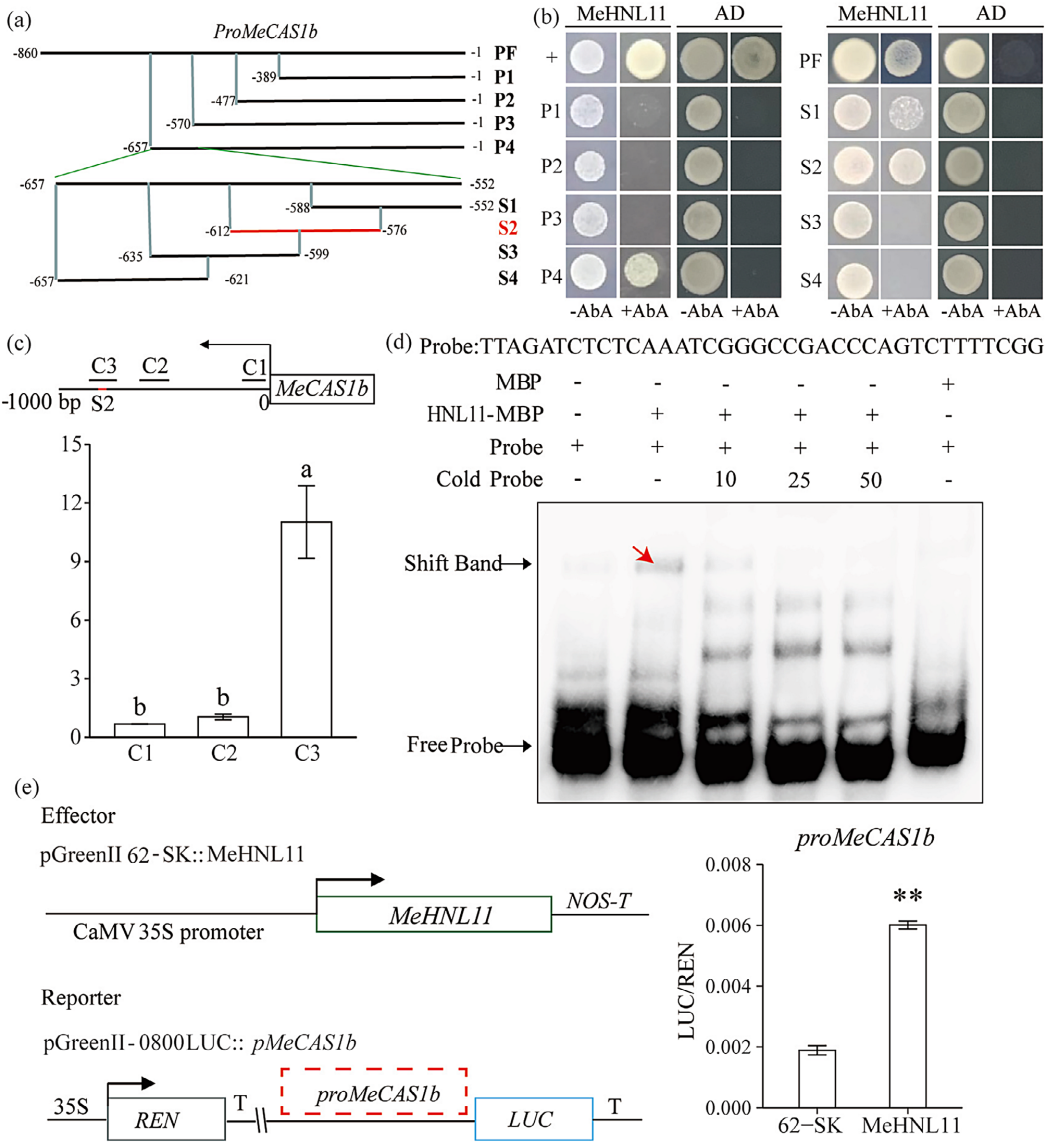
The binding sites determination of MeHNL11 on *MeCAS1bpro* in vivo and in vitro. (a) Distribution of truncated fragments in the upstream 860 bp promoter of *MeCAS1b*. (b) The interaction between AD‐MeHNL11 and *MeCAS1bpro* in yeast as determined by Y1H assay. (c) The MeHNL11‐GFP fusion protein was significantly enriched in the C3 region of the MeCAS1b promoter (the S2 site localised within the C3 region). (d) EMSA demonstrating MeHNL11 binding to S2 segment of the *MeCAS1bpro*. The S2 segment are noted in red. The position indicated by the red arrow represents the retarded band. (e) Transcriptional activation of *MeCAS1bpro* by MeHNL11 in protoplasts as measured by dual‐luciferase assays. Significance is denoted as *p* < 0.01 (**).

To elucidate the regulatory role of MeHNL11 on *MeCAS1b* under low N stress, a series of pAbAi bait vectors were constructed. These vectors incorporated either the full‐length *MeCAS1b* promoter (PF: 860 bp) or the truncated fragments (P1‐P4). Subsequently, these constructs were tested alongside the prey vector (MeHNL11‐AD) in the Y1H assay. The results demonstrated that only PF (−860 bp to 0) and P4 (−670 to −552) exhibited interaction with MeHNL11, indicating that the binding site is likely located in the −670 to −552 bp region. To further narrow down this binding site, we subdivided the −670 to −552 bp region into 4 segments (S1–S4), and conducted additional Y1H assay with MeHNL11. The results indicated that MeHNL11 bound to the S1 (−588 to −552 bp) and S2 (−612 to −576 bp) segments, with a notably stronger binding affinity observed for the S2 segment (Figure 6a,b).

To ascertain whether MeHNL11 can bind to the promoter of *MeCAS1b* in vivo, we conducted a chromatin immunoprecipitation (ChIP) assay on cassava protoplasts transfected with the 35S:HNL11‐GFP construct. qPCR primers were designed to amplify the segments C1‐C3 of the *MeCAS1b* promoter (Figure 6c, Table S2). The ChIP‐qPCR results indicated that there was a significant enrichment of the C3 region within the immunoprecipitated DNA, suggesting that MeHNL11 indeed interacts with the *MeCAS1b* promoter in vivo. Moreover, as illustrated in the Figure 6c, the S2 segment is located within the C3 region, further supporting our inference that MeHNL11 truly binds to the S2 segment in vivo.

The S2 segment was further used as a DNA probe for the Electrophoretic Mobility Shift Assay (EMSA) assay. When MeHNL11‐MBP was incubated with the S2‐labelled probe, a retarded band indicative of a DNA‐protein complex was observed. Moreover, as the concentration of the competitive probe increased, the intensity of the retarded band progressively diminished (Figure 6d). This suggests that MeHNL11 is capable of binding to the S2 segment in vitro.

The dual‐luciferase system was further employed to determine the regulatory effect of MeHNL11 on *MeCAS1b*. It was observed that both LUC luminescence and the LUC/REN ratio in cells co‐transfected with 62‐SK‐MeHNL11 and *MeCAS1bpro* were significantly elevated compared to those in the control group (Figure 6e). This indicates that MeHNL11 positively regulates the transcriptional activity of *MeCAS1b*. In summary, MeHNL11 not only binds to the promoter of *MeCAS1b* but also modulates its transcriptional activity. Consequently, it can be inferred that MeHNL11 exhibits the complete characteristics of a transcription factor.

### 2.7 The Monomeric Form of MeHNL11 Exhibits Enhanced Transcriptional Activation Capacity

**FIGURE 7:**
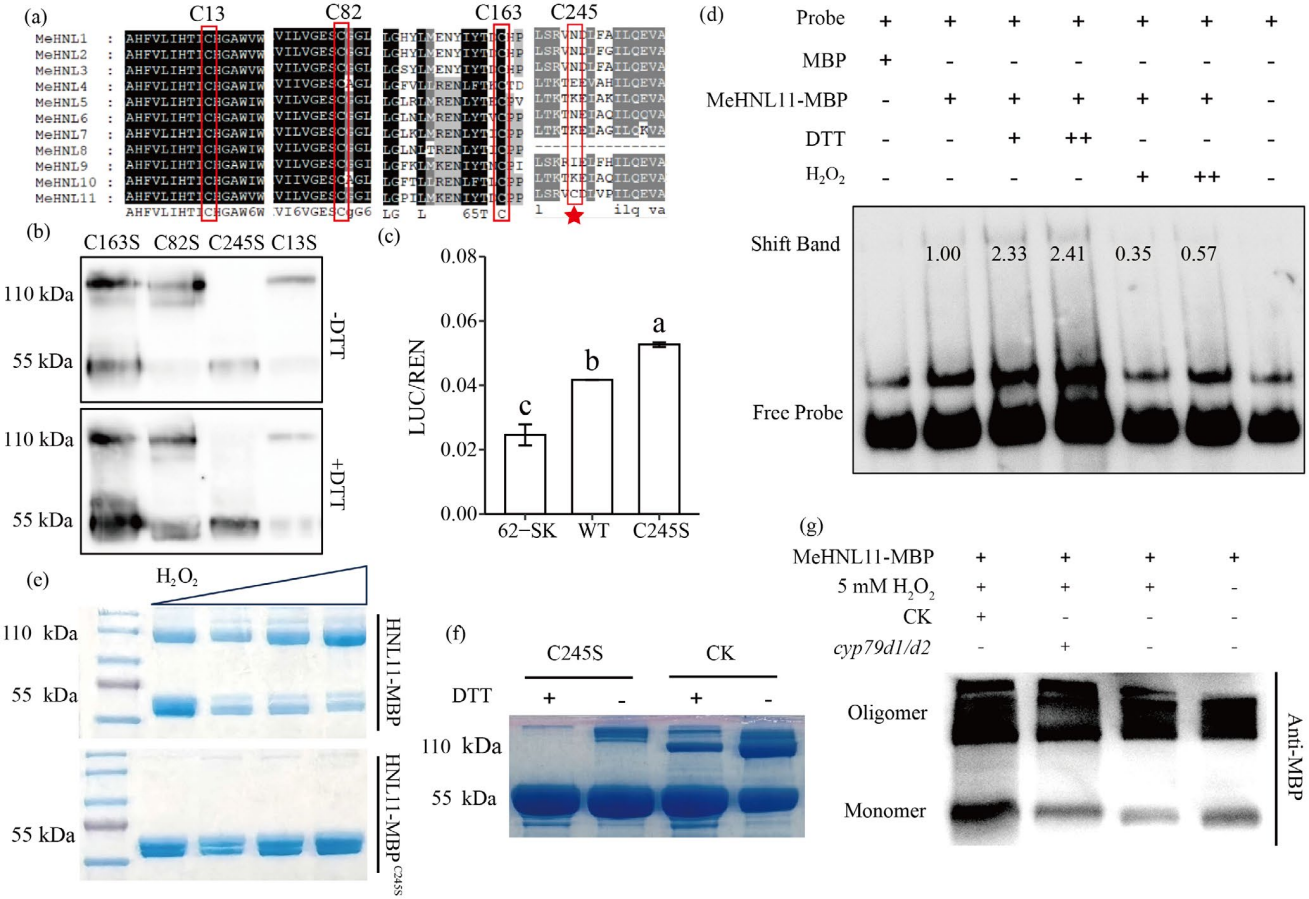
Comparison of the transcriptional activation capacity of MeHNL11 monomers and oligomers. (a) Amino acid sequence alignment of MeHNL11 and other MeHNL family members. (b) C245 is the key amino acid residue for MeHNL11 to form dimer determined by point mutation and DTT treatments. (c) Transcriptional activation of *MeCAS1bpro* by MeHNL11 and MeHNL^C245S^ in cassava protoplasts through dual‐luciferase assay. (d) The difference of MeHNL11‐MBP binding to the *MeCAS1bpro‐S2* between H_2_O_2_ and DTT pretreatment. (e) The dimerization state of MeHNL11‐MBP was enhanced by oxidant H_2_O_2_ treatment. (f) The dimerization state of MeHNL11‐MBP was reduced by the reductant DTT treatment. (g) The crude proteins from CK induced more pronounced monomerization of MeHNL11‐MBP than those from *cyp79d1/d2* mutants.

The MeHNL11 contains four cysteine residues (Cys13, Cys82, Cys163 and Cys245). Among these, Cys13, Cys82 and Cys163 are highly conserved within the MeHNLs family, whereas Cys245 is unique to MeHNL11 (Figure 7a). To investigate the role of these cysteines in MeHNL11 dimerization, point mutation expression vectors for each cysteine site were constructed and transiently expressed in tobacco (Figure 7b). Results showed that mutation of the unique Cys245 residue completely dissociated MeHNL11 into monomers, while mutation of the other cysteine sites could further dissociate MeHNL11 oligomers into monomers after DTT treatment, indicating that Cys245 is the critical residue mediating MeHNL11 dimer formation.

To compare the DNA‐binding ability of monomeric and oligomeric forms of MeHNL11, electrophoretic mobility shift assays (EMSA) were performed using a biotin‐labelled S2 segment of the *MeCAS1b* promoter as a probe and recombinant MeHNL11‐MBP protein pretreated with either DTT or H_2_O_2_ (Figure 7d–f). The results demonstrated that DTT treatment, which promotes the monomeric state of MeHNL11, significantly enhanced its binding to the S2 segment. In contrast, H_2_O_2_ treatment, which favours oligomer formation, markedly reduced its DNA‐binding affinity. Furthermore, a dual‐luciferase assay confirmed that the transcriptional activation capacity of the MeHNL11^C245S^ mutant was significantly stronger than that of wild‐type MeHNL11 (Figure 7c). Together, these data indicate that the monomeric form of MeHNL11 possesses stronger transcriptional activation capacity compared to its oligomeric forms.

The above studies have shown that cyanide could induce upregulation of *MeCAS1b* gene expression. To examine whether cyanide is also involved in regulating the monomerization of MeHNL11, crude protein extracts from wild‐type (CK) and *cyp79d1/d2* double mutant leaves were incubated with H_2_O_2_‐pretreated MeHNL11‐MBP recombinant protein (Figure 7g). Western blotting results revealed that crude extracts from CK induced more pronounced monomerization of MeHNL11‐MBP compared to those from the *cyp79d1/d2* mutant, suggesting that endogenously produced cyanide promotes the conversion of MeHNL11 from oligomeric forms to monomers, thus inducing the upregulation of *MeCAS1b*.

## 3 Discussion

### 3.1 MeHNL11 Is a Hydroxynitrile Lyase With Transcriptional Regulatory Activity

Growing research indicates that during the evolutionary process of life in both animals and plants, certain dual‐function proteins have evolved to more effectively maintain cellular metabolic homeostasis. These proteins not only possess enzymatic catalytic functions but also participate in the transcriptional regulation of specific genes. The involvement of these dual‐function proteins in transcriptional regulation can generally be categorised into two types. The first type pertains to modulating chromatin state to influence the transcription of target genes. For instance, inositol phosphate synthase (MIPS) can interact with methyltransferases ATXR5/6. This interaction maintains an active chromatin state within the *MIPS1* gene region, thereby facilitating its normal transcription (Latrasse et al. 2013). Similarly, methionine adenosyltransferase (MAT3) interacts with DNA topoisomerase VI to define chromatin boundaries to safeguard the expression of euchromatic island genes embedded within heterochromatic regions (Méteignier et al. 2022). The second type involves regulating transcription by directly binding to the promoters of target genes. Ornithine synthetase Arg5/6 binds to the promoters of *COX1*, *YOR352W*, and *PUF4* in response to limited N sources, but the transcription levels of these genes are significantly reduced in *arg5/6* mutants (Hall et al. 2004). Ceramide synthase (Schlank) binds to the promoter of lipase genes, regulating gene expression to adapt to the cell's energy state (Sociale et al. 2018). These examples vividly underscore the versatility of dual‐function proteins in regulating cellular metabolism and gene expression, as well as their key roles in ensuring cell survival across diverse environmental conditions.

In cyanogenic plants, HNL serves as a crucial defence against herbivore invasion by decomposing cyanohydrins to release highly toxic hydrogen cyanide (HCN). Initially, HNL was thought to primarily operate within the cytoplasm (Thayer and Conn 1981), but recent findings have unveiled that beyond its well‐known role in cyanohydrin decomposition, HNL may also play a part in transcriptional regulation. For example, AtHNL has been found to be distributed in both the cytoplasm and the nucleus (Arnaiz et al. 2022). In this study, MeHNL11 was identified to bind to the *MeCAS1b* promoter within the region spanning −612 bp to −576 bp, and was demonstrated to possess transcriptional activation ability. The binding capacity to the *MeCAS1b* promoter was further validated in vivo and in vitro (Figure 5). Significantly, when exposed to low nitrate stress, the localization of MeHNL11 in the nucleus accounts for a higher proportion. Based on these findings, it is proposed that MeHNL11 functions as a cyanohydrin catabolic enzyme while simultaneously exerting transcriptional regulation on its target gene, *MeCAS1b*.

### 3.2 MeHNL11 Forms Oligomers and Is Sequestered in the Nucleus Upon Low N Stress

Transcription factors generally have a nuclear localization signal (NLS), which guides their entry into the nucleus after being synthesized in the cytoplasm (Boulikas 1994). However, some transcription factors are retained in the cytoplasm under normal conditions and rapidly translocate to the nucleus in response to specific environmental stimuli, thereby precisely regulating the transcription activity of specific genes. This mechanism is closely associated with phosphorylation/dephosphorylation modifications (Jans and Hübner 1996). Previous studies have shown that upon NO_3_^−^ exposure, the NRT1.1‐CNGC15 module promotes the influx of Ca^2+^ (Liu et al. 2017), activating CPKs to phosphorylate the core N responsive regulatory genes like NLP7 (Wang et al. 2021), and facilitating their nuclear translocation.

In this study, we discovered that low N promotes the accumulation of MeHNL11 in the nucleus. MeHNL11 can form homodimers through disulfide bonds in the cytoplasm. But in the nucleus, in addition to dimers, higher‐order MeHNL11 polymers are formed. This oligomeric form may facilitate the retention of MeHNL11 within the nucleus. We are particularly interested in the mechanisms underlying the nuclear sequestration of MeHNL11 from three aspects: phosphorylation modification, formation of complexes with other proteins, and post‐translational modification by its product HCN. Previous studies reported that ACC oxidase can be modified by S‐cyanylation (García et al. 2019), which generates HCN by cleaving ACC (Houben and Poel 2019). Similarly, as an cyanide producing enzyme, MeHNL11 itself may also undergo S‐cyanylation modification. Such modification might potentially promotes its entry into the nucleus by altering the conformation of MeHNL11. Additionally, it remains to be investigated whether MeHNL11 enters the nucleus directly as a dimer or first disassembles into monomers.

### 3.3 The C245 and L29 Mutations Confer Dual Functionality of MeHNL11 as a Metabolic Enzyme and a Transcriptional Regulator

As previous studies have indicated that cassava MeHNL family members typically function as oligomers (Hughes et al. 1994; White et al. 1998; Chueskul and Chulavatnatol 1996), our study also found that MeHNL11 primarily exists as oligomers. However, the formation of dimers/polymers depends on its unique cysteine residue at position 245 (C245). Mutation at this site causes MeHNL11 to completely disassemble into monomers and significantly enhances its transcriptional activation ability. On one hand, the C245 site is required for its normal enzymatic function; on the other hand, it may act as a molecular switch for speeding up its transcriptional regulation activity, enabling MeHNL11 to respond to cyanide or low N stress signals, thereby facilitating the transition from oligomers to monomers and achieving precise regulation of *MeCAS1b* transcriptional activity. Additionally, MeHNL11 harbours a unique leucine residue at position 29 (L29); this residue may contribute to the formation of a leucine zipper‐like structure, which could confer DNA‐binding activity on MeHNL11 (Noman et al. 2017; Perotti et al. 2017), thus enabling the monomer to also possess transcription factor‐like function. Taken together, the presence of C245 and L29 endows MeHNL11 with dual functions as both a metabolic enzyme and a transcription factor.

### 3.4 MeHNL11 Is a HCN Producer and a Sensitive Responder to HCN Signals, Initiating the Detoxification of HCN Under N Deficiency

HCN has the ability to bind to cytochrome oxidase C, thereby inhibiting respiration and ultimately resulting in cellular hypoxia and death. However, it is noteworthy that all plants are capable of producing HCN. Increasing evidence suggests that low concentrations of HCN act as a signalling molecule to regulate multiple physiological processes (Díaz‐Rueda et al. 2023). Given the toxicity of HCN, plants are in urgent need of acutely sensing changes in HCN concentration within cells and promptly maintaining it at a non‐toxic level. In fact, it has been observed that a wide range of abiotic stresses can cause an increase in cyanide content in plants. In response, plants rapidly upregulate the transcriptional levels of cyanoalanine synthase (CAS) to prevent excessive accumulation of cyanide (Liang 2003; Machingura et al. 2013; Xu et al. 2023), but the underlying molecular mechanism remains elusive.

In this research, we observed a significant upregulation of *MeCAS1b* under low N stress. However, when plants were treated with the cyanide detoxifier COB, this upregulation was significantly attenuated. Furthermore, in the *cyp79d1/d2* mutants, the expression of *MeCAS1b* failed to increase effectively in response to low N stress (Figure 1e). These findings imply that low N stress might promote the degradation of CGs, leading to the accumulation of cyanide, and that elevated cyanide levels are responsible for the enhanced expression of *MeCAS1b*. At the same time, the elevated cyanide enters the nucleus and induces the dissociation of the MeHNL11 oligomers into monomers, and there was a strong correlation between the nuclear proportion of MeHNL11 and the expression levels of *MeCAS1b* (Figures 1e and 4). In the *cyp79d1/d2* mutants, the nuclear accumulation of MeHNL11 was significantly reduced and did not further increase under low N stress (Figure 3). Based on these observations, we propose that MeHNL11 might be the direct sensor of cyanide signals, which likely modulates the transcriptional activity of *MeCAS1b* to maintain HCN concentration at an appropriate level in cassava plants.

### 3.5 Cyanogenic Glycosides Act as an Organic N Pool Responsive to Low N Stress

Cassava is a crop rich in CGs, which were initially primarily known as defensive compounds against herbivore feeding. However, when plants encounter N deficiency, CGs can be rapidly mobilised and converted into primary N metabolites to satisfy the N‐related demands of the plant. This phenomenon has been reported in sorghum, rubber, eucalyptus and flax (Gleadow and Woodrow 2000; Kongsawadworakul et al. 2009; Blomstedt et al. 2018; Siegien et al. 2021). In cassava, compared to the wild type, *CYP79D2* RNAi lines display significantly enhanced nitrate reductase activity in roots, and exhibit stunted growth and develop curled leaves when cultured in ammonium‐deficient medium (Siegien et al. 2021; Siritunga and Sayre 2003). Additionally, overexpression of CG‐degrading enzymes or cyanide assimilating enzymes has been demonstrated to increase the content of proteins and free amino acids in cassava roots (Narayanan et al. 2011; Leyva‐Guerrero et al. 2012). These findings indicate that CGs serve as an important reduced‐N reservoir in cassava.

This study found that under low N treatment, the synthesis genes of CGs in cassava are suppressed, while the degradation genes are activated. This results in a reduced accumulation of CGs. In contrast, when N is abundant, the synthesis of CGs is upregulated to enhance cassava's resistance to herbivores. This indicates that cassava can rewire the transcriptional profile of the CG metabolic pathway in response to the availability of external N. Take together, CGs represent a more economical defensive strategy, not only protecting against herbivores but also strengthening the N reserve pool. This could be one of the reasons why cassava is tolerant to infertile soils.

The cyanoalanine pathway is the most widely reported route for the reuse of CGs, but its impact on the N metabolism of cassava under low N stress remains unclear. In this study, by using COB treatment to block the cyanide assimilation mediated by MeCAS1b, it was found that compared with low N treatment alone, the NH_4_^+^ and AA contents in cassava significantly decreased, and the N‐deficiency symptoms became more prominent when subjected to both low N and COB treatments. This suggests that the cyanide assimilation mediated by MeCAS1b is an important N source supplement for cassava during low N adaptation.

Compared to the NN treatment, cassava seedlings in the NNC treatment also exhibited growth retardation; therefore, the potential effects of the COB treatment on plants need to be considered. The COB treatment contains cobalt (Co) atoms, and high concentrations of Co can interfere with chlorophyll synthesis and lead to chlorosis in young leaves (Lange et al. 2017). However, in the LNC and NNC treatments, the main symptoms were yellowing and abscission of older leaves, while young leaves did not show chlorosis. This phenotypic pattern is inconsistent with the typical symptoms of Co toxicity. Thus, the primary cause of growth retardation in the LNC and NNC treatments is likely N deficiency rather than Co toxicity induced by the COB treatment.

Current studies have shown that the leaf linamarase and hydroxynitrile lyase are located in the cell wall rather than the cytoplasm (Mkpong et al. 1990; White et al. 1998), but the cyanide assimilation capacity in roots is significantly higher than that in leaves (Tawanda et al. 2017), which implies that the reuse of CGs in cassava may occur primarily in the roots. This study found that under normal N conditions, *MeCAS1b* is mainly expressed in leaves. However, when exposed to low N conditions, the upregulation of *MeCAS1b* in roots becomes more obvious. Additionally, unlike other MeHNL members that are mainly expressed in leaves, MeHNL11 is specifically expressed in roots and is induced by low N, suggesting that the MeHNL11‐*MeCAS1b* module may primarily mediate the reuse of CGs in roots under low N stress. Previous studies have demonstrated through girdling experiments that CGs in cassava are mainly synthesised in leaves and transported to roots via the phloem, with leaves serving as the main storage site for CGs (Jørgensen et al. 2005). Therefore, it is hypothesized that under low N stress, a larger quantity of CGs in leaves is transported to roots and recycled through the cyanoalanine synthesis pathway in roots, highlighting a dynamic adjustment in the allocation and utilisation of N within cassava.

### 3.6 MeHNL11‐ *MeCAS1b* Module Recycles CGs to Combat Low N Stress

In conclusion, this study proposes a hypothesis regarding the reuse of CGs under low N conditions. It is postulated that low N stress triggers the transport of CGs from leaves to roots, where CGs are decomposed by BGLU and HNL to generate HCN. This HCN production promotes the dissociation of MeHNL11 oligomers into monomers in the nucleus, which then sharply activates the transcription of *MeCAS1b*. Subsequently, HCN is converted into asparagine, aspartic acid and ammonium under the sequential catalysis of MeCAS1b and MeNIT4, thus supplying the plant with the essential N for growth and development. This process illustrates a sophisticated mechanism by which cassava can recycle N from CGs to cope with N‐deficient environments (Figure S3). It uncovers a novel dimension of N use efficiency in cassava under LN.

## 4 Materials and Methods

### 4.1 Plant Material and Treatments

South China 8 (SC8) Cassava plantlets were cultivated in greenhouse conditions in Haikou, Hainan, China. The growth conditions were set as a 14‐h light/10‐h dark photoperiod, with a daytime temperature of 28°C and a nighttime temperature of 26°C. After 20 days, SC8 plantlets were exposed to either N deficiency (0 mM) or normal N levels (5 mM NO_3_^−^ or 5 mM NH_4_^+^) for another 20 days period.

### 4.2 RNA‐Seq

After extraction of total RNA from the sample, mRNA is enriched and then reverse transcribed to generate double‐stranded cDNA. In the subsequent steps, the double ends of the cDNA are repaired, adapters are added and PCR amplification is carried out to construct a library. Fastp is utilised to filter out low‐quality data in accordance with predefined criteria, thus yielding clean reads. These clean reads are aligned to the *Manihot esculenta* v8.1 genome using bowtie2 and HISAT2 software (Shi et al. 2021). The FPKM (Fragments Per Kilobase of transcript per Million) value for each gene was calculated based on the original read count, adjusted for the length of the gene or transcript. Then the differential expression results are presented in the form of a volcano plot. To identify significantly enriched pathways among the differentially expressed genes relative to the entire gene set, a hypergeometric test is conducted with the KEGG pathway as the unit of analysis (He et al. 2020).

### 4.3 Yeast One‐Hybrid Assay (Y1H)

The promoter of *MeCAS1b*, spanning the 860 bp upstream regions from the transcription start codon, was cloned into pAbAi vector to serve as bait. A full‐length SMART III cDNA library was constructed using root tissues from N‐deficient seedlings treated with a 0.5 mM NO_3_^−^ concentration (Wei et al. 2024). To determine the minimum AbA concentration for screening, fusion vector pAbAi‐*MeCAS1bpro* was transformed into Y1H Gold yeast. Subsequently, the cells were spread on SD/‐Ura‐Leu plates containing various AbA concentrations (0, 50, 100, 200, 300, 400, 500, 600 ng/mL). Simultaneously, the cDNA library was transferred into the Y1H Gold yeast containing *MeCAS1bpro*. The diluted cells (100 fold) were spread on SD/‐Ura‐Leu plates to assess the efficiency of Y1H assay. For screening of interacted proteins, the remaining cells were spread on SD/‐Ura‐Leu plates (AbA = 400 ng/mL). After secondary streaking on SD/‐Ura‐Leu plates with AbA = 400 ng/mL, individual clones were picked for PCR identification and Sanger sequencing. The sequence data obtained from Sanger sequencing were aligned with the reference cassava genome for annotation.

### 4.4 Yeast Two‐Hybrid Assay (Y2H)

The pGBKT7‐MeHLN11 construct was generated to verify the transcriptional activation ability of MeHLN11 using Y2H assay. Following the Y2HGold chemical conversion method (Sha et al. 2024), pGBKT7‐MeHLN11 and pGADT7 were co‐transformed into Y2HGold yeast strain. The BD‐53 + AD‐p53 combination was used as positive control, while BD‐lam + AD served as negative control. Subsequently, the co‐transformed yeast cells were serially diluted and spotted onto selective media: SD/−Trp‐Ade, SD/−Trp‐His and SD/−Trp‐Ade‐His + X‐α‐Gal. This step enabled the assessment of MeHNL11's transcriptional activation ability. By observing growth and colour changes on the different media, we could determine whether transcriptional activation occurred or not.

### 4.5 Protoplast Preparation From Cassava Leaf

For protoplast extraction, the leaves of 1‐month‐old SC8 cassava plantlets were tenderly cut into 1 mm thin strips. These strips were then immersed in a 0.9% mannitol solution and incubated in the dark for 30 min. Subsequently, the leaf strips were transferred into a 50 mL conical flask containing 10 mL of enzymatic solution. Enzymatic hydrolysis was carried out at 45 rpm in the dark for 16 h. After hydrolysis, the protoplasts were washed twice with W5 solution, then the purified protoplasts were diluted to a concentration ranging from 1 × 10^5^ to 5 × 10^5^/mL using MMG solution, preparing them for plasmid transfection (Wu et al. 2017).

### 4.6 Subcellular Localisation

The coding sequence (CDS) of *MeHNL11* was cloned into G1300‐GFP vector, which is driven by CaMV 35S promoter. The recombinant vector G1300‐MeHNL11‐GFP, along with the control vector G1300‐GFP, was transfected into cassava mesophyll protoplasts using a polyethylene glycol (PEG)‐mediated method as described in Hsu et al. (2010). Green fluorescent signals were observed using laser scanning confocal microscopy (Olympus FV3000, Japan).

The nuclear area was delineated using DAPI staining, and cytoplasm defined as total GFP fluorescent signal minus nuclear area. To analyse the distribution of fluorescence between the nucleus and the cytoplasm, the mean fluorescence intensity (MFI) ratio of nuclear to cytoplasmic area per cell was determined and quantified using the ImageJ ‘Intensity Ratio Nuclei Cytoplasm Tool’. The results were plotted as MFI (nucleus/cytoplasm), following the approach described by Hilleary et al. (2020).

### 4.7 Dual‐Luciferase Reporter Assay

The *MeCAS1b* promoter was inserted into pGreen‐0800 vector to serve as reporter plasmid. To construct the effector plasmid, the coding sequence (CDS) of *MeHNL11* was inserted into the pGreen‐62‐SK vector. The plasmids pGreen‐0800‐*MeCAS1b* and pGreen‐62‐SK‐MeHNL11 were co‐transfected into cassava mesophyll protoplasts and cultured in the dark for 16 h. A control group was also set up by co‐transfecting pGreen‐0800‐*MeCAS1b* and pGreen‐62‐SK without the *MeHNL11* insert. The LUC/REN value was measured using a Luciferase Assay System kit (Promega, Waldorf, Germany) according to the manufacturer's instructions (Zhang et al. 2018; Liu et al. 2023).

### 4.8 Electrophoretic Mobility Shift Assays (EMSAs)

EMSA was performed as previously reported (Bao et al. 2024). The MeHNL11‐MBP recombinant protein was expressed and purified following the method described in Wang et al. (2020). The probe was incubated with the purified MeHNL11 protein at 25°C for 30 min. Subsequently, the mixture was loaded into the well of a 5% polyacrylamide gel, and electrophoresis was conducted at 100 V under low‐temperature conditions. After electrophoresis, the separated components were transferred onto membranes and cross‐linked. Subsequently, the biotin‐labelled probe was visualised using a chemiluminescence method with the GelDoc imaging system (BIO‐RAD, Hercules, California, USA).

### 4.9 Chromatin Immunoprecipitation (ChIP)

ChIP was performed using the G1300‐MeHNL11 recombinant plasmid, which was transfected into cassava mesophyll protoplasts. The cells were then cross‐linked with 1% formaldehyde for 10 min and the reaction was terminated with glycine. After sonication to shear the chromatin complex, the BeyoChIP ChIP Assay Kit was employed for subsequent experiments. Immunoprecipitation was conducted using anti‐GFP antibodies to selectively capture the MeHNL11‐GFP fusion protein‐DNA complexes, with anti‐IgG antibodies serving as a negative control. After reversal of cross‐linking and protein digestion, the DNA was purified using a PCR purification kit to obtain the immunoprecipitated DNA for further analysis (Hong et al. 2021).

### 4.10 Western Blotting

Protein samples were resolved using SDS‐polyacrylamide gel electrophoresis, and then transferred to a PVDF membrane. A 5% skim milk solution was employed to block the membrane, preventing non‐specific antibody binding. Specific antibodies were applied to immunoreact with their target proteins on the membrane. Finally, the protein was detected using a chemiluminescence method, which allows for the visualisation of the protein bands on the membrane (Shi et al. 2021).

### 4.11 Subcellular Fractionation

Subcellular fractionation was performed as previously described, using protoplast from SC8 cassava plantlet (Wu et al. 2017). Isolated proteins were treated with 10 mM DTT to disrupt the disulfide bonds. The proteins were then fractionated by Western blot as described earlier, using a GFP antibody to detect the MeHNL11‐GFP fusion. An anti‐α‐actin antibody was used for the cytoplasmic fraction and an anti‐Histone H3 antibody for nuclear fractions, respectively. This procedure allowed for the assessment of MeHNL11‐GFP distribution between cytoplasm and nucleus, providing insights into its subcellular localization.

### 4.12 Quantitative Real‐Time PCR (RT‐qPCR)

Total RNA extraction, cDNA synthesis and RT‐PCR were carried out as described in Zheng et al. (Zheng et al. 2024). For RT‐PCR, each sample was run in quadruplicate, and *β‐actin* (Manes.13G084300) was used as internal control. Relative expression of target genes was calculated by the 2^−ΔCT^ method. Statistical significance was assessed using One‐way ANOVA and Tukey's test with SPSS9.2 software. Primers used for RT‐qPCR are listed in Table S2.

## Author Contributions

C.Z., X.C. and W.W. conceived and supervised the project. W.M., R.B., X.L., C.Z. and X.C. wrote and revised the manuscript. W.M., M.L., and X.L. performed the experiments. J.Z., H.Z. and H.W. participated in the experimental assay, phenotype measurements and data analysis. Y.Y. provided *cyp79d1/d2* mutant.

## Funding

This work was supported by National Natural Science Foundation of China (32572475, 32260509). The China Agriculture Research System (CARS11‐HNCX). Hainan Province Science and Technology Special Fund (ZDYF2023XDNY179). Project of State Key Laboratory of Tropical Crop Breeding (NKLTCBCXTD07). Hainan University Research Star‐up Funding (KYQD20016).

## Conflicts of Interest

The authors declare no conflicts of interest.

## Supporting information


**Figure S1:** The transcription factor screening of *MeCAS1bpro* with Yeast one‐Hybrid (Y1H). (a) Concentration of Aureobasidin A (AbA) utilised for Y1H screening with pAbAi‐*MeCAS1bpro*. At the concentration of 400 ng/mL AbA, it effectively suppress the background growth of Y1H strains containing pAbAi‐*MeCAS1bpro* on SD/‐Ura plates; (b) Conversion efficiency assessment for the Y1H screening of pAbAi‐*MeCAS1bpro*. 10^−2^: The Y1H strains with pAbAi‐*MeCAS1bpro* were diluted a factor of 10 times and spread on SD/‐Ura/−Leu plates; (c) Reconfirmation of yeast cloning. Monoclonal yeast clones from Y1H assay were purified by streaking on SD/‐Ura/−Leu plates containing AbA = 400 μg/mL. (d) Verification of pAbAi‐*MeCAS1bpro* Y1H screening results by PCR. The purified monoclonal clones were subjected to PCR, and the resulting PCR products are used for subsequent sequencing analysis.

**Figure S2:** Assessment of the transcription by MeHNL11‐GFP using Y2H assay. ‘positive control’: pGBKT7D‐p53 + pGADT7‐T; ‘negative control’: pGBKT7‐lam + pGADT7‐T; SD/−TA: Double Dropout Medium (Lacking Tryptophan and Adenine); SD/‐TH: Double Dropout Medium (Lacking Tryptophan and Histidine); SD/‐TAH: Triple Dropout Medium (Lacking Tryptophan, Adenine and Histidine). X‐α‐Gal: X‐α‐D‐Galactoside.

**Figure S3:** The pathway of cyanogenic glycosides response to nitrogen deficiency. (left) Red‐doted square represent the synthesis of cyanogenic glycosides (CGs); Blue‐doted square indicate decomposition of CGs; Blue‐doted square referr to the cyanide base assimilation associated pathway. (right) Model of MeHNL11 functions as a transcriptional factor to regulate the transcriptional activity of *MeCAS1b* under N Deficiency. Under nitrate starvation, the expression of *MeCYP79D2* is downregulated, Meanwhile, the CGs stored in the vacuole are transported to the roots, where they are decomposed by emzyme MeHNL11 to produce cyanide. The accumulated cyanide triggers MeHNL11 to form oligomers and translocate into the nucleus to regulate the expression of *MeCAS1b*. This transcriptional activation of *MeCAS1b*, enabling the convert organic nitrogen of CGs into reusable primary metabolites of nitrogen, thus enhancing the adaptability of cassava to low nitrogen environments.

**Table S1:** The proteins interacted with promoter of *MeCAS1b* screened by Y1H assay.

**Table S2:** Primer pairs used in this study.

## Data Availability

The data that supports the findings of this study are available in the Supporting Information S1 of this article.
