# Supplementary figures and images for: Metabolic Enzyme MeHNL11 Regulates MeCAS1b Transcription for Cyanide Reutilization in Response to Nitrate Deficiency in Cassava
Source: Plant Biotechnol J. 2026 Mar 10;24(6):4121–35. doi: 10.1111/pbi.70633 (PMC13205736; doi:10.1111/pbi.70633)

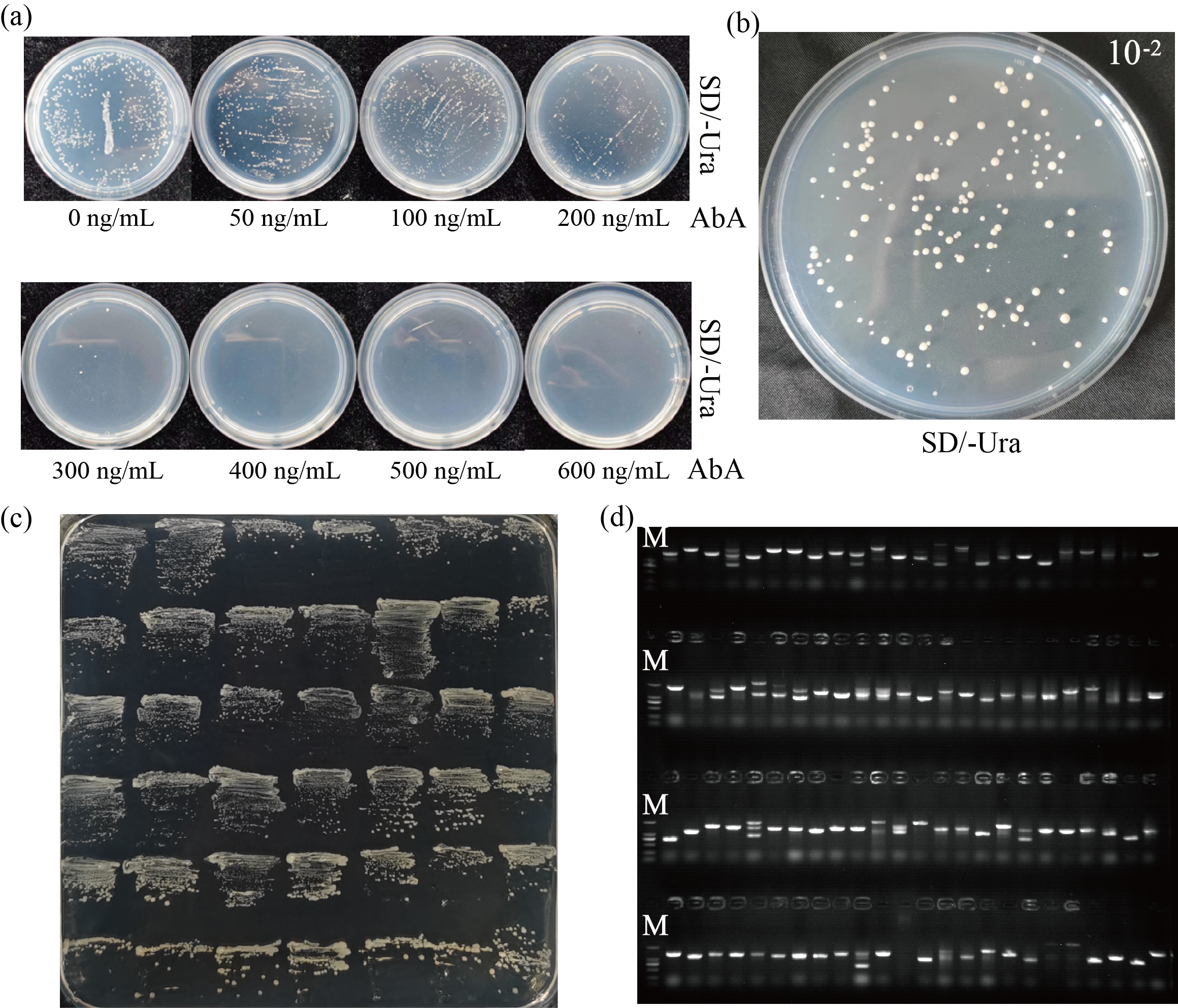

Supplement: Supplementary file 1 — Figure S1: The transcription factor screening of MeCAS1bpro with Yeast one‐Hybrid (Y1H). (a) Concentration of Aureobasidin A (AbA) utilised for Y1H screening with pAbAi‐MeCAS1bpro. At the concentration of 400 ng/mL AbA, it effectively suppress the background growth of Y1H strains containing pAbAi‐MeCAS1bpro on SD/‐Ura plates; (b) Conversion efficiency assessment for the Y1H screening of pAbAi‐MeCAS1bpro. 10−2: The Y1H strains with pAbAi‐MeCAS1bpro were diluted a factor of 10 times and spread on SD/‐Ura/−Leu plates; (c) Reconfirmation of yeast cloning. Monoclonal yeast clones from Y1H assay were purified by streaking on SD/‐Ura/−Leu plates containing AbA = 400 μg/mL. (d) Verification of pAbAi‐MeCAS1bpro Y1H screening results by PCR. The purified monoclonal clones were subjected to PCR, and the resulting PCR products are used for subsequent sequencing analysis. [file PBI-24-4121-s003.tif]

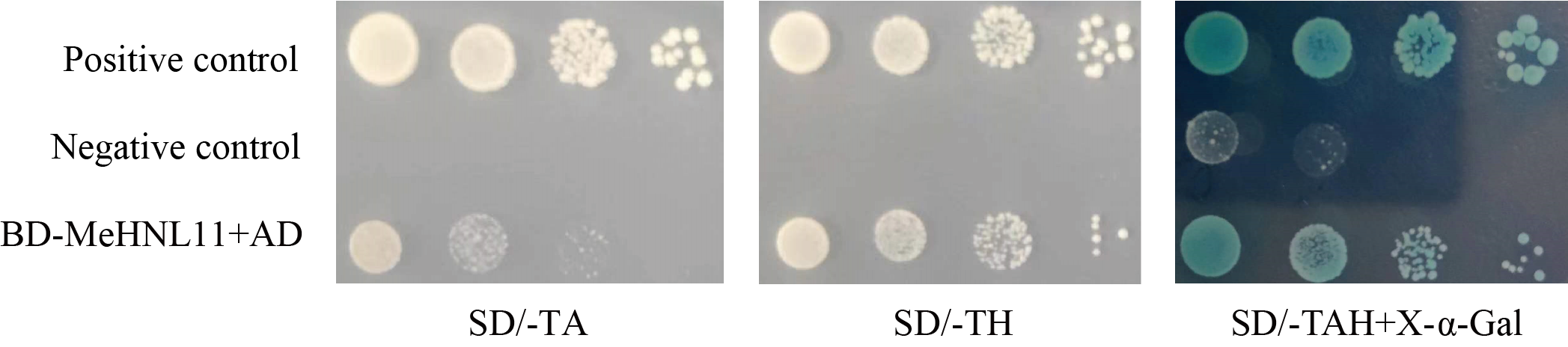

Supplement: Supplementary file 2 — Figure S2: Assessment of the transcription by MeHNL11‐GFP using Y2H assay. ‘positive control’: pGBKT7D‐p53 + pGADT7‐T; ‘negative control’: pGBKT7‐lam + pGADT7‐T; SD/−TA: Double Dropout Medium (Lacking Tryptophan and Adenine); SD/‐TH: Double Dropout Medium (Lacking Tryptophan and Histidine); SD/‐TAH: Triple Dropout Medium (Lacking Tryptophan, Adenine and Histidine). X‐α‐Gal: X‐α‐D‐Galactoside. [file PBI-24-4121-s002.tif]

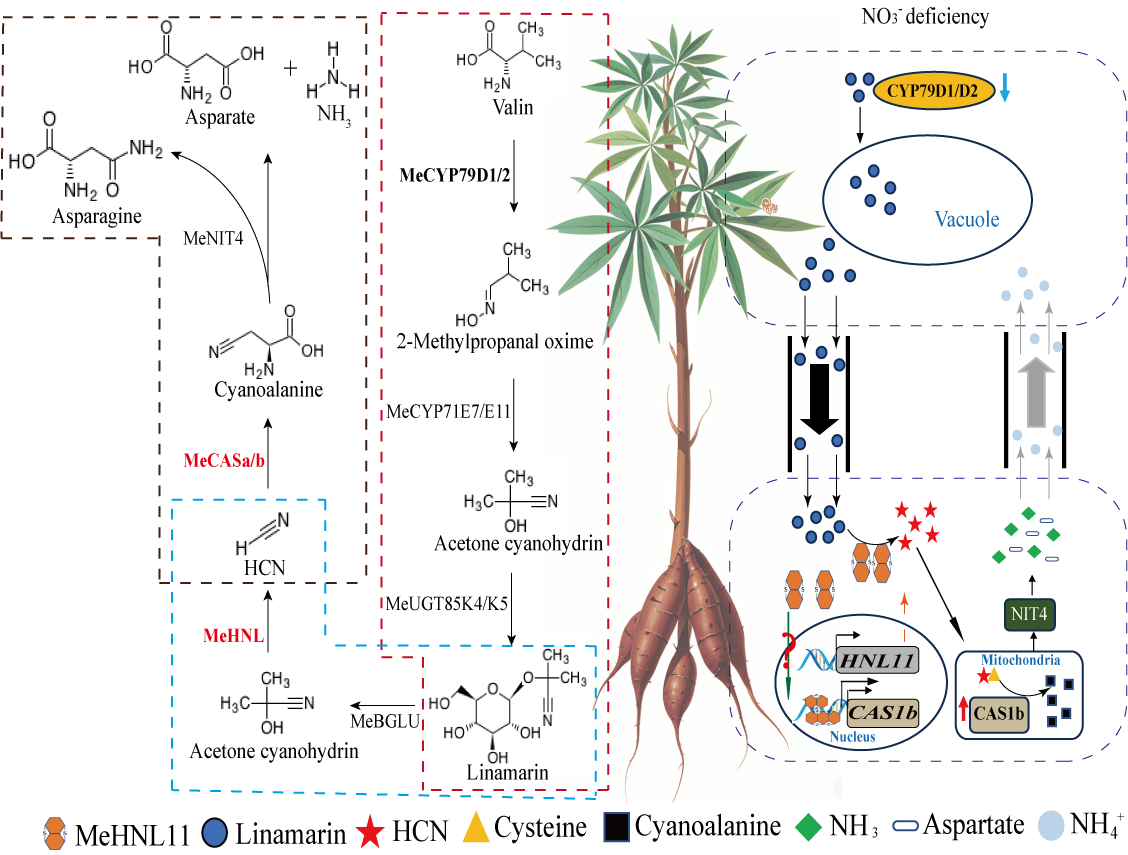

Supplement: Supplementary file 3 — Figure S3: The pathway of cyanogenic glycosides response to nitrogen deficiency. (left) Red‐doted square represent the synthesis of cyanogenic glycosides (CGs); Blue‐doted square indicate decomposition of CGs; Blue‐doted square referr to the cyanide base assimilation associated pathway. (right) Model of MeHNL11 functions as a transcriptional factor to regulate the transcriptional activity of MeCAS1b under N Deficiency. Under nitrate starvation, the expression of MeCYP79D2 is downregulated, Meanwhile, the CGs stored in the vacuole are transported to the roots, where they are decomposed by emzyme MeHNL11 to produce cyanide. The accumulated cyanide triggers MeHNL11 to form oligomers and translocate into the nucleus to regulate the expression of MeCAS1b. This transcriptional activation of MeCAS1b, enabling the convert organic nitrogen of CGs into reusable primary metabolites of nitrogen, thus enhancing the adaptability of cassava to low nitrogen environments. [file PBI-24-4121-s001.tif]
